# Effects of bacterial inoculants on the indigenous microbiome and secondary metabolites of chamomile plants

**DOI:** 10.3389/fmicb.2014.00064

**Published:** 2014-02-19

**Authors:** Ruth Schmidt, Martina Köberl, Amr Mostafa, Elshahat M. Ramadan, Marlene Monschein, Kenneth B. Jensen, Rudolf Bauer, Gabriele Berg

**Affiliations:** ^1^Institute for Environmental Biotechnology, Graz University of TechnologyGraz, Austria; ^2^Faculty of Agriculture, SEKEM, Heliopolis University, Ain Shams UniversityCairo, Egypt; ^3^Department of Pharmacognosy, Institute of Pharmaceutical Sciences, University of GrazGraz, Austria; ^4^Institute of Chemistry, University of GrazGraz, Austria

**Keywords:** bioactive secondary metabolites, biological control agents, chamomile, microbial communities, soil-borne pathogens

## Abstract

Plant-associated bacteria fulfill important functions for plant growth and health. However, our knowledge about the impact of bacterial treatments on the host's microbiome and physiology is limited. The present study was conducted to assess the impact of bacterial inoculants on the microbiome of chamomile plants *Chamomilla recutita* (L.) Rauschert grown in a field under organic management in Egypt. Chamomile seedlings were inoculated with three indigenous Gram-positive strains (*Streptomyces subrutilus* Wbn2-11, *Bacillus subtilis* Co1-6, *Paenibacillus polymyxa* Mc5Re-14) from Egypt and three European Gram-negative strains (*Pseudomonas fluorescens* L13-6-12, *Stenotrophomonas rhizophila* P69, *Serratia plymuthica* 3Re4-18) already known for their beneficial plant-microbe interaction. Molecular fingerprints of 16S rRNA gene as well as real-time PCR analyses did not show statistically significant differences for all applied bacterial antagonists compared to the control. In contrast, a pyrosequencing analysis of the 16S rRNA gene libraries revealed significant differences in the community structure of bacteria between the treatments. These differences could be clearly shown by a shift within the community structure and corresponding beta-diversity indices. Moreover, *B. subtilis* Co1-6 and *P. polymyxa* Mc5Re-14 showed an enhancement of the bioactive secondary metabolite apigenin-7-*O*-glucoside. This indicates a possible new function of bacterial inoculants: to interact with the plant microbiome as well as to influence the plant metabolome.

## Introduction

Whereas large-scale efforts have rapidly advanced the understanding of plant genomes, the impact and importance of the plant's microbiome is largely unexplored. Comparable to the human microbiome, millions of microbes inhabit plants, forming a complex ecological community that influences plant growth and health through its collective metabolic activities and host interactions (Berg, 2009; Lugtenberg and Kamilova, 2009). Currently, the many studies on plant-associated microorganisms reflect the full effect of ongoing research and the enormous interest in this topic (Mendes et al., 2011, 2013; Berendsen et al., 2012; Bakker et al., 2013). Viewing the microbiota from an ecological perspective could provide insight into how to promote health and stress tolerance of their hosts or how to adapt to a changing climate by targeting this microbial community. Furthermore, new functions of the plant microbiome can be detected. Several studies revealed that rhizobacteria have an effect on the aroma of fruits, e.g., strawberry and grapes (Pirlak and Köse, 2009; Verginer et al., 2010). Moreover, induced systemic resistance has been triggered in several crops by plant growth promoting bacteria (Murphy et al., 2003; Kloepper et al., 2004; Ryu et al., 2004a,b). Interestingly, certain plant growth-promoting rhizobacteria (PGPR) elicit induced systemic resistance and plant growth promotion in the absence of physical contact with plants via volatile organic compound (VOC) emissions (Farag et al., 2013). In addition, selected PGPR strains have been shown to reduce disease in plant parts through the induction of defense compounds, and especially members of the endomicrobiome have been shown to be involved in the production of bioactive compounds (Pimentel et al., 2011; Gutierrez et al., 2012). For several medicinal plants, bacteria and fungi were detected as producers of their active ingredients. Paclitaxel and maytansine, known as important anticancer lead molecules, were detected to be produced by endophytes (Chandra, 2012; Wings et al., 2013). Traditional Chinese Medicine is using indigenous medicinal plants integrated in an ancient healing system originating almost 4500 ago. For several of those plants it was shown that they are active due to its microbial endophytes (Miller et al., 2012; Zhao et al., 2012). However, these discoveries are only the beginning of understanding the complex interactions between plants and microbes, and new omics technologies will promote this.

Promotion of plant health or biological control is one of the well-studied functions of the plant microbiome. It is based on naturally occurring antagonists and offers sustainable solutions for plant protection (Weller, 2007; Berg, 2009; Raaijmakers et al., 2009). Gram-negative bacteria, especially those from the genus *Pseudomonas*, were identified as the dominant members of the indigenous antagonistic communities under humid conditions (Berg et al., 2005a; Haas and Defago, 2005; Costa et al., 2006; Zachow et al., 2008). In addition, these strains were also identified as a major group of disease-suppressive bacteria through pyrosequencing (Mendes et al., 2011). In contrast, under arid conditions, we found mainly Gram-positive bacteria as antagonistic counterparts (Köberl et al., 2011). To verify this finding as well as to find out which bacterial strains—indigenous Gram-positive strains in comparison with allochthonous Gram-negative strains—support plant growth under arid conditions in desert farming, different bacterial inoculants were developed and tested under field conditions.

The overall aim of this study was to evaluate the impact of several bacterial inoculants on the indigenous microbial community of chamomile plants and the production of bioactive secondary metabolites. We selected three Gram-positive strains isolated in Egypt and three European Gram-negative strains already known for their beneficial plant-microbe interactions and used as biocontrol agents (Lottmann and Berg, 2001; Zachow et al., 2010). Bacterial inoculants were applied to chamomile plants [*Chamomilla recutita* (L.) Rauschert] grown under field conditions with organic (biodynamic) management on Sekem farms in Egypt, and the impact of the treatment on the indigenous microbial communities was monitored. This is important to understand the potential risk of biocontrol but also to understand the mode of action of bacterial inoculants, which can be mediated by the plant microbiome as well (Scherwinski et al., 2008).

## Results

### Chemical analysis of chamomile secondary metabolites

**Figure 1:**
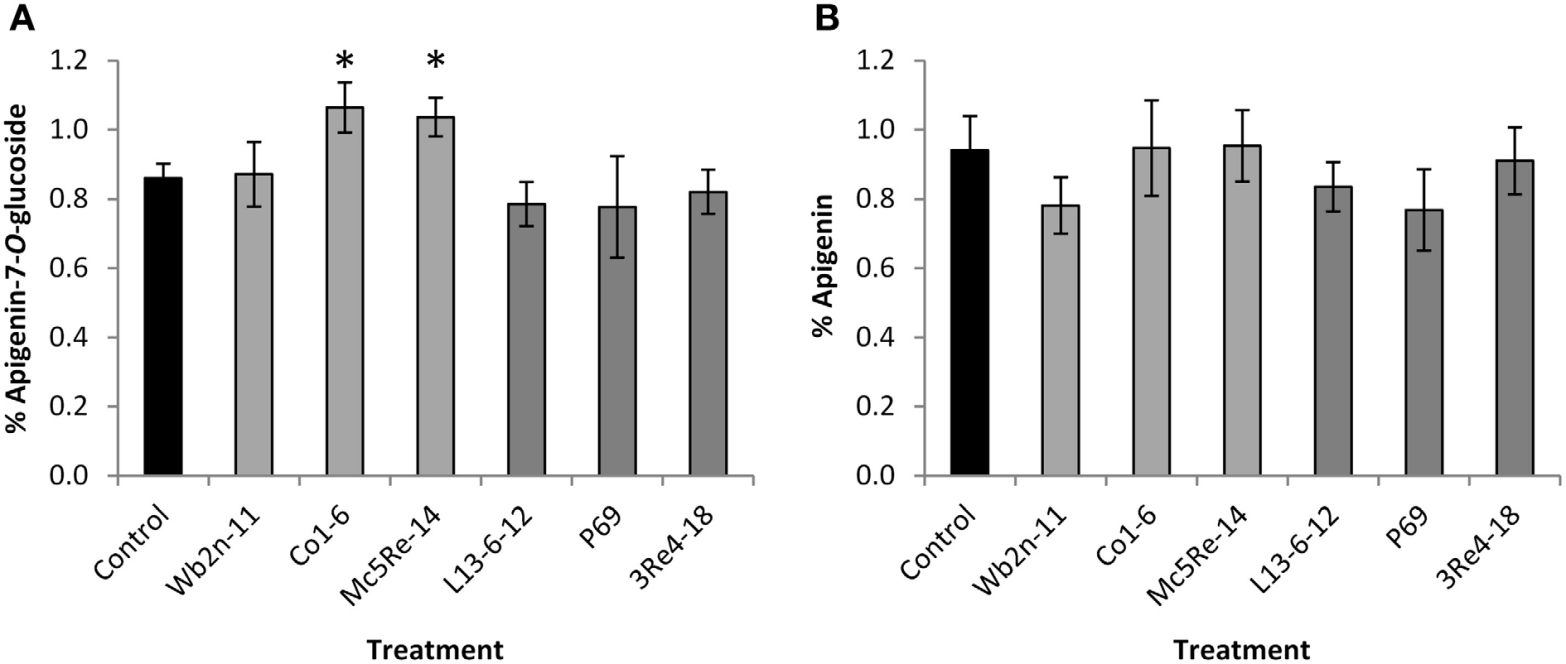
**Content (%) of apigenin-7-*O*-glucoside (A) and apigenin **(B)** in *Chamomilla recutita* (L.) Rauschert samples**. Averages of individual HPLC-MS measurements and confidences are shown. Significant differences (*p* < 0.05) of bacterial treatments (*Streptomyces subrutilus* Wb2n-11, *Bacillus subtilis* Co1-6, *Paenibacillus polymyxa* Mc5Re-14, *Pseudomonas fluorescens* L13-6-12, *Stenotrophomonas rhizophila* P69, *Serratia plymuthica* 3Re4-18) to the control are indicated by asterisks.

HPLC-MS experiments of the flower extracts yielded slightly different contents of apigenin-7-*O*-glucoside and apigenin between the different treatments (Figure 1). For apigenin-7-*O*-glucoside, statistically significant higher contents than for the non-inoculated control plants (0.86 ± 0.04) were observed for the treatments with the indigenous Gram-positive strains *Bacillus subtilis* Co1-6 (1.06% ± 0.07) and *Paenibacillus polymyxa* Mc5Re-14 (1.04% ± 0.06). The autochthonous *Streptomyces subrutilus* Wb2n-11 (0.87% ± 0.09) and all three allochthonous Gram-negative strains *Pseudomonas fluorescens* L13-6-12 (0.79% ± 0.06), *Stenotrophomonas rhizophila* P69 (0.78% ± 0.15), and *Serratia plymuthica* 3Re4-18 (0.82% ± 0.06) showed no elevation of apigenin-7-*O*-glucoside content (Table S1). Highest contents of apigenin were obtained for treatments with the Gram-positive strains *B. subtilis* Co1-6 (0.95% ± 0.14) and *P. polymyxa* Mc5Re-14 (0.95% ± 0.10) as well, however without significant difference to the control (0.94% ± 0.10). Also *P. fluorescens* L13-6-12 (0.84% ± 0.07), *S. rhizophila* P69 (0.77% ± 0.12), *S. plymuthica* 3Re4-18 (0.91% ± 0.10), and *S. subrutilus* Wb2n-11 (0.78% ± 0.08) did not show an alteration (Table S2). Investigation of peaks which arose in the treatments with the two autochthonous Bacillales strains (m/z = 327 and 329 for [M-H]^−^) resulted in the possible identification of [C_18_H_32_O_5_ – H^+^]^−^ (Rt: 13.15 min) and [C_18_H_34_O_5_ – H^+^]^−^ (Rt: 14.8 min), respectively.

### Molecular fingerprinting of the chamomile-associated bacterial communities

**Figure 2:**
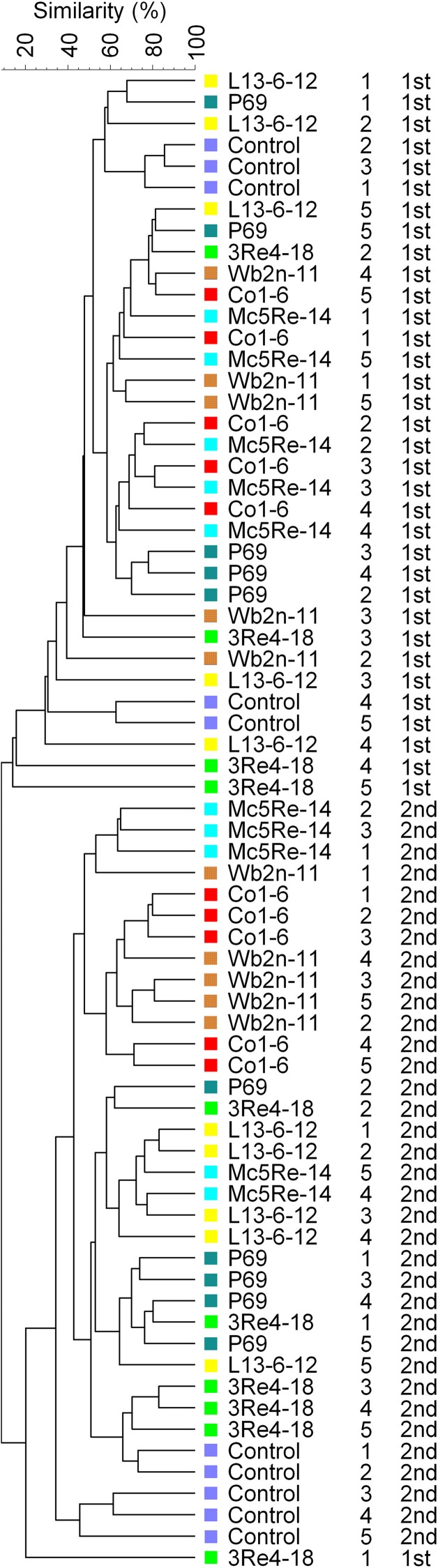
**Cluster analysis of eubacterial community fingerprints**. Similarities between SSCP fingerprints were calculated using the curve-based Pearson correlation coefficient and grouped according to their similarity using the hierarchical UPGMA. Treatments (*Streptomyces subrutilus* Wb2n-11, *Bacillus subtilis* Co1-6, *Paenibacillus polymyxa* Mc5Re-14, *Pseudomonas fluorescens* L13-6-12, *Stenotrophomonas rhizophila* P69, *Serratia plymuthica* 3Re4-18, and water control) and sampling times (1st) after 4 weeks and (2nd) after 8 weeks are indicated. Numbers 1–5 mark the five independent replicates per treatment.

**Figure 3:**
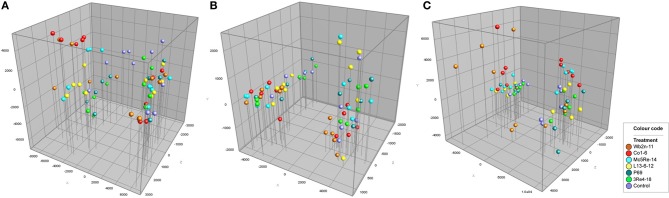
**Principal component analysis (PCA) of eubacterial (A), *Pseudomonas* (B) and *Firmicutes* (C) community fingerprints**. Treatments (*Streptomyces subrutilus* Wb2n-11, *Bacillus subtilis* Co1-6, *Paenibacillus polymyxa* Mc5Re-14, *Pseudomonas fluorescens* L13-6-12, *Stenotrophomonas rhizophila* P69, *Serratia plymuthica* 3Re4-18, and water control) are indicated by colors. Sampling time (1st) after 4 weeks is shown on the left side and (2nd) after 8 weeks is shown on the right side. PCA was calculated based on relative positions and intensity of DNA bands.

In the molecular fingerprinting approach, universal primers were used to get a first overview about the whole bacterial community associated with *Chamomilla recutita* (L.) Rauschert as well as *Pseudomonas*- and *Firmicutes*-specific primers. Community composition was determined by image analysis of the band profiles generated by single-stranded conformational polymorphism (SSCP) analysis, and differences in the bacterial community composition based on these band patterns were calculated using the Pearson's correlation index. Cluster analysis of the microbial profiles resulted in grouping of rhizosphere samples at different sampling times at about 10% similarity (Figure 2). These differences between the two sampling times were confirmed by a principal component analysis (Figure 3). Within each cluster, only several of the samples from the different treatments were grouped together. Generally, samples from different treatments were found to be more similar to each other than the samples of the five replicates of each specific treatment, suggesting no significant differences arising from the bacterial inoculants. Moreover, no cluster group was found containing exclusively SSCP patterns of one specific treatment.

The bacterial community associated with *C. recutita* (L.) Rauschert showed a high abundance of *Bacillus* spp. (Table S3). In the *Pseudomonas* community two dominant bands could be detected, which were abundant in both sampling times. These two bands were identified by partial 16S rRNA gene sequence analysis as *Pseudomonas* sp. (closest database match *Pseudomonas* sp. MOC14, 100% similarity to JX122114.1) (Table S4). In the *Firmicutes* community *Bacillus* spp. were found in all samples, whereas *Bacillus* sp. BMR7, 99–100% similarity to JX434152.1 and *Bacillus* sp. DV9-6, 99% similarity to GQ407151.1, represented the most abundant species (Table S5).

### Pyrosequencing-based 16S rRNA profiling of chamomile-associated bacterial communities

**Figure 4:**
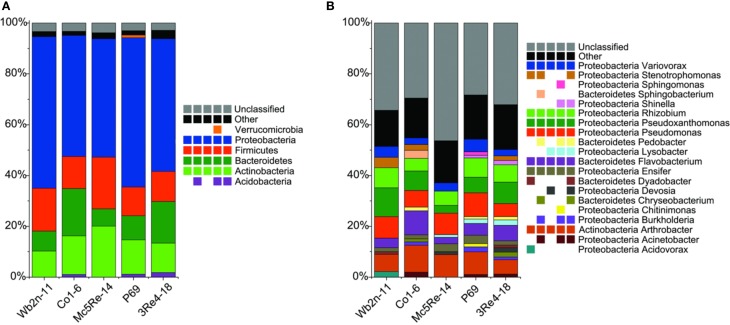
**Classification of bacterial communities associated with *Chamomilla recutita* (L.) Rauschert**. Pyrosequencing reads were classified at phylum **(A)** and genus **(B)** level against RDP core set within QIIME pipeline with an 80% confidence threshold. Taxa below 1% of relative abundance are included in “Other.” Multi-colored charts at the legend are shown for each sample correspondingly.

**Figure 5:**
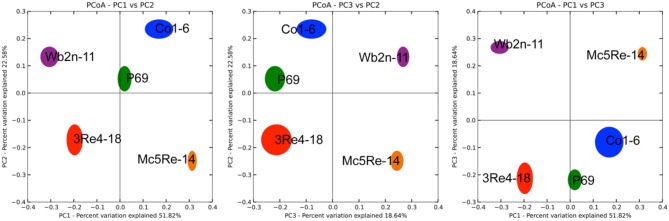
**Comparison of the microbial communities of *Chamomilla recutita* (L.) Rauschert rhizosphere by jackknifed principal coordinate analysis**. The 2D-plot illustrates the compositional similarity between samples based on weighted UniFrac. The positions of the points are the averages for the jackknifed replicates generated by QIIME and are shown with ellipses representing the interquartile range (IQR) in each axis. Larger ellipses represent more diverse communities. Colors correspond to the different treatments.

**Figure 6:**
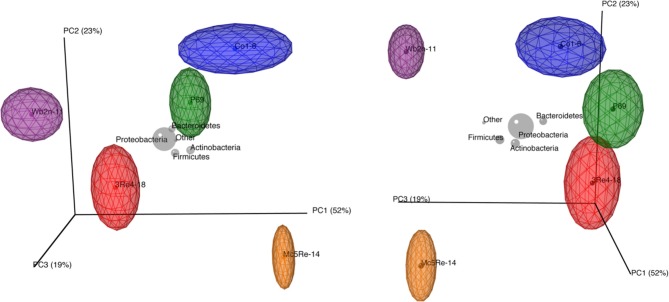
**Comparison of the microbial communities of *Chamomilla recutita* (L.) Rauschert rhizosphere by jackknifed principal coordinate analysis**. The biplot illustrates the compositional similarity between samples based on weighted UniFrac. Taxa coordinates are indicated by gray orbs with size, as a function of relative abundance. To confine the biplot, the number of the displayed taxa was restricted to 5. The positions of the points are the averages for the jackknifed replicates generated by QIIME and are shown with ellipses representing the interquartile range (IQR) in each axis. Larger ellipses represent more diverse communities. Colors correspond to the different treatments.

To deeply investigate the diversity and the composition of the bacterial communities associated with *C. recutita* (L.) Rauschert, a pyrosequencing approach was employed. Rarefaction analysis was performed to an extent of diversity coverage (Figure S1). Assessment of richness revealed that pyrosequencing effort attained 35.8-46.5% of estimated richness at a genetic similarity of 97% (Table 1). At the genetic similarity levels of 95% and 80%, amplicon libraries covered 41.7–49.7% and 56.6–88.8% of estimated richness, respectively (Table 1). Taxonomic composition of bacterial communities was similar at phylum level, comprising *Proteobacteria*, *Bacteroidetes*, *Firmicutes*, and *Actinobacteria* as the most dominant phyla (Figure 4). However, the phylum *Verrucomicrobia* was only present in the sample from the treatment with *S. rhizophila* P69, considering only taxa covering more than 1% of quality sequences. *Acidobacteria* were observed in samples treated with *B. subtilis* Co1-6, *S. rhizophila* P69, and *S. plymuthica* 3Re4-18. At genus level, *Rhizobium* (phylum Proteobacteria), *Pseudoxanthomonas* (phylum Proteobacteria), *Pseudomonas* (phylum Proteobacteria), *Flavobacterium* (phylum *Bacteroidetes*), and *Arthrobacter* (phylum *Actinobacteria*) represented the most abundant genera, showing a different composition according to the different treatments. Alpha-diversity of the amplicon libraries was characterized by Shannon index (H') for 97, 95, and 80% similarity levels. Slight differences between treatments where revealed by the comparison of the index values (Table 1). Jackknifed weighted UniFrac two-dimensional (Figure 5) and three-dimensional (Figure 6) principal coordinates analysis (PCoA) biplots were constructed in order to visualize relationships among samples based on differences in taxonomic diversity. Weighted biplots showed that the samples were clearly separated, implying a difference in bacterial community composition according to the treatments.

**Table 1:** **Richness estimates and diversity indices for amplicon libraries of rhizosphere samples^a^**.

**Genetic similarity^b^**	**Sample^c^**	**No. of OTUs**	**Chao1**	**Coverage (%)**	**H'^d^**
97%	Wb2n-11	557	1443	38.6	5.14
	Co1-6	596	1280	46.5	5.32
	Mc5Re-14	729	1928	37.8	5.66
	P69	559	1209	46.2	5.23
	3Re4-18	546	1525	35.8	5.37
95%	Wb2n-11	433	871	49.7	4.80
	Co1-6	477	998	47.8	5.00
	Mc5Re-14	560	1342	41.7	5.26
	P69	435	930	46.8	4.90
	3Re4-18	419	973	43.1	5.01
80%	Wb2n-11	81	143	56.6	2.85
	Co1-6	76	86	88.8	3.05
	Mc5Re-14	90	134	67.4	2.87
	P69	92	120	76.9	3.11
	3Re4-18	89	100	88.6	3.20

a *The number of sequences of each sample was normalized to 1858*.

b *Genetic similarities represent the taxonomic levels of species (97%), genera (95%), and phyla (80%)*.

c *Abbreviations correspond to the treatments with Streptomyces subrutilus (Wb2n-11), Bacillus subtilis (Co1-6), Paenibacillus polymyxa (Mc5Re-14), Stenotrophomonas rhizophila (P69), and Serratia plymuthica (3Re4-18)*.

d *Shannon diversity indices*.

### Quantitative analysis of bacterial abundances

The bacterial abundances of total bacteria and *Firmicutes* were determined by quantitative PCR. For total bacteria, abundances ranged from 7.45 log_10_ copies per g root fresh weight (fw) for the treatment with *S. subrutilus* Wb2n-11 to 8.03 log_10_ copies per g fw for the treatment with *P. polymyxa* Mc5Re-14 (Figure S2). Abundances for *Firmicutes* ranged from 6.97 log_10_ copies per g fw for the treatment with *S. subrutilus* Wb2n-11 to 7.54 log_10_ copies per g fw for the treatment with *P. polymyxa* Mc5Re-14 (Figure S2). However, no statistically significant differences between the treatments could be detected.

**Figure 7:**
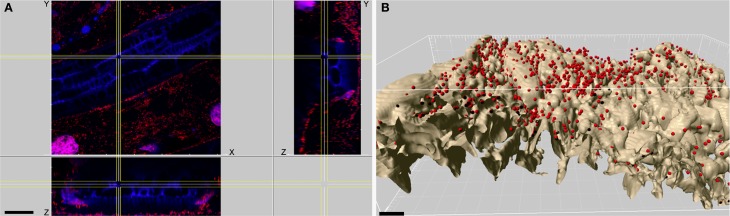
**Root epidermis of a chamomile seedling 14 days after inoculation**. Visualization of DsRed marked bacteria with confocal laser scanning microscopy. **(A)** xy, xz, and yz maximum projections showing a colonizing of small colonies and single cells of *Serratia plymuthica* 3Re4-18 of the surrounding area of the root. Red: bacterial cells, blue: root, scale bar: 30 μm. **(B)** Surface model of **(A)** shows the root-surface localization of *Serratia plymuthica* 3Re4-18 in the three-dimensional space. Red: bacterial cells, brown: root, scale bar: 10 μm.

The colonization pattern of the labeled bacterial (DsRed) strain *S. plymuthica* 3Re4-18 was monitored with confocal laser scanning microscopy. For chamomile seedlings cultivated in a gnotobiotic system, cells of *S. plymuthica* 3Re4-18 were able to colonize the rhizosphere. (Figure 7A). Two different colonization patterns could be observed: single cells, covering the root surface and forming a dense network. Furthermore, cells were often found as surrounding clouds (Figure 7B), where they were loosely arranged around the root surface.

## Discussion

In this study, we examined the impact of six bacterial inoculants on the native bacterial community of *C. recutita* (L.) Rauschert as well as on the production of flavonoid compounds. We found an impact of the treatments on both, host's microbiome and physiology.

The impact of bacterial inoculants was analyzed by three methods. No impact was found at quantitative level: all abundances were highly similar and showed no statistically significant differences. Quantitative insight into the microbial communities in the rhizosphere showed that total bacteria (up to 8.03 log_10_ copies per g root fw for the treatment with *P. polymyxa* Mc5Re-14) as well as *Firmicutes* (up to 7.54 log_10_ copies per g fw for the treatment with *P. polymyxa* Mc5Re-14) resulted in a high abundance in the rhizosphere. The phenomenon that the number of microorganisms in the rhizosphere is enhanced as a result of exudation of compounds by the root was described as the rhizosphere effect by Lynch (1990). However, no clear influence on the abundance of microorganisms by bacterial inoculants could be detected, even though results give a hint that Gram-positive bacteria are more dominant in the rhizosphere than introduced Gram-negative strains. The high abundance of Gram-positive bacteria in bulk soil was already described by Smalla et al. (2001).

At qualitative level, the applied methods resulted in different conclusions. All molecular fingerprints obtained with universal and group-specific primers revealed high similarity of microbial community composition and were strongly related to the sampling times, showing about 10% similarity. In both sampling times, the bacterial rhizosphere community did not differ between the treatments and the control. The rhizosphere of the young chamomile plant was shown to be colonized mainly by *Bacillus* spp. and *Pseudomonas* spp. The high abundance of *Bacillus* species in the field soil at Sekem farms was already described by Köberl et al. (2011). Pseudomonads represent important members of the rhizosphere microbial community due to their aggressive colonization (Scherwinski et al., 2006). Their plant growth promoting ability was described in several studies (Bloemberg and Lugtenberg, 2001; Weller, 2007). Generally, the species composition differed slightly between the two sampling times. However, due to the limited number of identified strains, these results should be interpreted carefully. The SSCP analysis showed that there was no clear influence by the bacterial inoculants on the diversity of the naturally occurring bacterial populations in the rhizosphere of *C. recutita* (L.) Rauschert. Next-generation sequencing allows a deeper insight into microbial community composition, and therefore answers to the following questions can be provided: (i) how are microbial communities composed on different taxonomic levels? (ii) how does the structure of communities look like between different samples? (iii) how do communities change across treatments? To address these questions, samples from different treatments with bacterial inoculants were investigated and compared among each other. At first, the pyrosequencing approach showed similar patterns of bacterial diversity between the different treatments, whereas *Proteobacteria*, *Actinobacteria*, *Acidobacteria*, *Chloroflexi*, *Verrucomicrobia*, *Bacteroidetes*, *Planctomycetes*, *Gemmatimonadetes*, and *Firmicutes* represented the most abundant phyla in all analyzed samples (>1% of all sequences). Janssen (2006) already described members of these identified phyla as the most abundant soil bacteria. The phylum *Verrucomicrobia* was only present in the sample from the treatment with *S. rhizophila* P69 (=DSM14405^T^). In other *ad planta* studies, this strain was found to have an indirect positive interaction with their host plants by altering fungal communities (Schmidt et al., 2012). When bacteria were analyzed at the genus level, the relative abundances of different genera belonging to phyla *Proteobacteria*, *Bacteroidetes*, and *Actinobacteria* varied among the samples under different treatments. These results showed that it is of crucial importance to be aware of using the appropriate levels of taxonomy for different investigations. Therefore, this study suggests that for the detection of differences within microbial communities deriving from bacterial inoculants the use of genus or even lower levels of taxonomy yield the greatest benefits. Unlike alpha diversity measurements, such as species richness and rarefaction curves, beta diversity measures the degree of similarity, e.g., phylogenetic relatedness between pairs of communities (Caporaso et al., 2010). Therefore, phylogenetic beta diversity metrics were used to investigate the structure of communities between the samples. PCoA plots showed that samples differed according to the treatments. Despite the SSCP results, pyrosequencing results suggest that the introduction of antagonistic bacterial strains leads to changes in the native bacterial community structure.

The impact on the physiology of the host was found for the treatments with the two Gram-positive strains *B. subtilis* Co1-6 and *P. polymyxa* Mc5Re-14, yielding a significantly higher content of apigenin-7-*O*-glucoside compared to the treatments with Gram-negative strains. Apigenin was only found in traces and may have been produced postharvest by hydrolysis from apigenin-7-*O*-glucoside (Bauer and Wagner, 1991; Maier et al., 1993). Likewise, previous studies have isolated and identified the presence of both mono- and di-acetylated apigenin-7-*O*-glucoside, which are known to undergo rapid ester hydrolysis leading to the formation of apigenin-7-*O*-glucoside (Redaelli et al., 1981; Svehlikova et al., 2004). Moreover, apigenin-7-*O*-glucoside is highly susceptible for the hydrolysis to its corresponding aglycone in presence of acid or hydrolytic enzymes. As a result, the formation or degradation of apigenin-7-*O*-glucoside may lead to a falsification of quantification. To date, only few studies have considered the influence of rhizobacteria on the secondary metabolites of plants (Zuanazzi et al., 1998; Singh et al., 2002; Orhan et al., 2006). Therefore, little is known about the mechanisms behind positive influence of rhizobacterial secondary metabolites on the plants secondary metabolite production. Plants have the ability to acquire enhanced level of resistance to pathogenic microorganisms, a mechanism first described by Van Peer et al. (1991) and Wei et al. (1991) as induced systemic resistance (ISR). However, ISR can also be induced by various non-pathogenic microorganisms that may activate inducible defense mechanisms in the plant in a similar way to pathogenic microorganisms (Loon, 2007). Activation of defense mechanisms by plants suggests that even a beneficial rhizobacterium may be recognized as a potential threat, leading to the production of resistance compounds (Kloepper et al., 2004; Ongena et al., 2005; Schuhegger et al., 2006; Arkhipova et al., 2007; Vleesschauwer and Höfte, 2007; Lehr et al., 2008; Choudhary and Johri, 2009; Lal and Tabacchioni, 2009).

Next-generation sequencing allows a deeper insight into plant-associated microbial communities. This was already shown for suppressive soils (Mendes et al., 2011) and for the analysis of core microbiomes (Lundberg et al., 2012). In our study we show that they also improve our understanding of the mode of interaction of bacterial inoculants. The latter are promising for a sustainable agriculture and the challenges for crop production in the context of climate change. To understand the effects and interactions of biocontrol agents will allow the improvement of biocontrol products.

## Materials and methods

The bacterial strains used in this study are summarized in Table 2. For the preparation of inoculums, several colonies of each bacterial strain were inoculated in 500 ml liquid LB medium (Roth, Karlsruhe, Germany) and grown at 30°C and 150 rpm for 24 h (Co1-6, L13-6-12, P69, and 3Re4-18) and for 48 h (Wb2n-11 and Mc5Re-14). To harvest the cells, bacterial suspensions were centrifuged at 13,500 g for 20 min. Pellets were dissolved in 2 ml sucrose solution (1%), serving as cryoprotectant agent, and frozen to −70°C for 5 h. Tubes containing the frozen bacterial suspension were put into ampules and connected to a freeze-dryer (Labconco FreeZone 4.5 Liter Benchtop, USA) for 12 h under vacuum at <0.1 Pa.

**Table 2:** **Bacterial strains used in this study**.

**Bacterial strain**	**Origin**	**References**
*Streptomyces subrutilus* Wb2n-11	Desert soil in Sinai, Gram-positive	Köberl et al., 2011
*Bacillus subtilis* subsp. subtilis Co1-6	Rhizosphere of *Calendula officinalis* (L.), Gram-positive	Köberl et al., 2011
*Paenibacillus polymyxa* Mc5Re-14	Endorhiza of *Chamomilla recutita* (L.) Rauschert, Gram-positive	Köberl et al., 2011
*Pseudomonas fluorescens* L13-6-12	Rhizosphere of *Solanum tuberosum* (L.), Gram-negative	Lottmann and Berg, 2001
*Stenotrophomonas rhizophila* P69 (= DSM14405^T^)	Rhizosphere of *Brassica napus* (L.), Gram-negative	Wolf et al., 2002
*Serratia plymuthica* 3Re4-18	Endorhiza of *Solanum tuberosum* (L.), Gram-negative	Berg et al., 2005b; Grosch et al., 2005

### Field experiment and sampling procedure

Prior to planting, one-month old field-grown *C. recutita* (L.) Rauschert seedlings were root-dipped in suspensions of the bacteria for 15 min. The non-treated control seedlings were dipped in tap water and planted. Five replicates of each treatment were performed in a randomized block design (1 × 2 m per plot) at a field at Adleya farm/Sekem (30°22′88″N; 31°39′41″E). During the growth stage, the field was irrigated with water (2607 l m^−3^ on average per year) coming from the Nile or from local groundwater drillings—drip irrigation systems were used. Composite soil samples were collected from each plot at a surface depth of 0–5 cm, and the soil-texture was classified as sandy silt with a pH of 8.48, 254.75 μ S/m electrical conductivity, 1.44% organic matter, 0.82% organic carbon, 0.15% total nitrogen, 0.01% total phosphorous, and 0.07% total potassium. Rhizosphere samples were taken at 4 and 8 weeks after planting, each with five replicates per treatment. A sample consisted of 5 g roots with adhering soil. In the end of the growing season (March 2012), chamomile flowers were harvested, each with five replicates per treatment.

### Determination of flavonoids

Fresh chamomile flowers were dried in a forced-convection oven at 40°C and milled to a fine powder using a universal IKA M 20 grinding mill (IKA-Werke, Staufen, Germany). The extraction of chamomile flower heads was performed using an accelerated solvent extractor, ASE 200 (Dionex Corporation, Sunnyvale, CA, USA). Extraction was carried out at a temperature of 68°C, with a constant pressure of 69 bar and a static time of 5 min using 100% methanol (v/v) as extraction solvent. Based on preliminary experiments, conditions of ASE 200 were set as follows: no preheating period, heating time of 5 min, flush volume at 30% of the extraction cell volume, three extraction cycles, nitrogen purge time of 60 s. The extracts were filtered (Whatman No. 42) and stored at 4°C in darkness until the chromatographic analysis. HPLC analysis was performed on an UltiMate 3000 RS chromatographic system (Dionex, Sunnyvale, CA, USA). A LiChrospher 100 RP-18 (125x4 mm, 5 μm) (Merck, Darmstadt, Germany) was employed for the separation. The binary mobile phase consisted of solvents A (water) and B (acetonitrile) according to the following profile: 0–17 min, 15–40% B; 17–18 min, 40–75% B; 18–29 min, 75% B. The flow rate was 1.0 ml/min and the injection volume was 5 μ l. Chromatograms were recorded at a wavelength of 340 nm. For the mass analysis, a linear ion trap quadrupole (LTQ) XL mass spectrometer (Thermo Scientific, San Jose, CA, USA) with an electrospray interface (ESI) was used. ESI negative ion mode conditions were set as follows: source voltage 3.0 kV, sheath gas flow rate 50 au, auxiliary gas flow rate 10 au, source current 100.0 μ A, capillary voltage −45.0 kV, and capillary temperature 330°C. The screening was performed in full scan, covering the range from m/z 50 up to 2000. Calibration curves of apigenin-7-O-glucoside and apigenin were obtained by the external standard method. Apigenin-7-O-glucoside and apigenin were quantified using Xcalibur Quan Browser (Version 2.0, Thermo Fisher, San Jose, CA, USA). Chromatograms of each treatment were overlaid with the control using MZmine (Version 2.8, Katajamaa et al., 2006) in order to identify new peaks arising from the treatments. Identification of new peaks was performed using a Dionex Ultimate 3000 UHPLC focused LC system coupled via a heated electrospray source HESI2 to a Q-Exactive mass spectrometer (Thermo Fisher Scientific). HPLC conditions were set as described above. HESI conditions were set as follows: spray voltage: −3 kV, capillary temp: 450°C, sheath and auxiliary gas: N2, flow: 75 & 20 instrument units, gas temp heater: 350°C. The MS instrument was operated in negative mode, externally mass calibrated, and with the resolving power sat to 140000 (FWHM), scanned between m/z 133-2000. With an exclusion time of 10 s, the five most intense ions were selected for collision induced fragmentation. The selection window was 0.4 Dalton, and the fragmentation energy NCE sat to 30 ± 20% instrument units. MS2 spectra were obtained with the resolving power sat to 35000. Putative compounds were identified using the online METLIN metabolite database (http://metlin.scripps.edu).

### Rhizosphere sampling and total community DNA isolation

The bacterial fraction associated with *C. recutita* (L.) Rauschert was extracted using the protocol adapted from Opelt and Berg (2004). In brief, for each rhizosphere sample, 5 g of roots with adhering soil were mixed with 45 ml NaCl solution (0.85%) and vortexed for 5 min. A total volume of 4 ml of the suspension was centrifuged at 16,000 g at 4°C for 20 min, and the pellet was used for isolation of the total community DNA. For mechanical lysis, the cells were homogenized in a FastPrep FP120 Instrument (MP Biomedicals, Solon, OH, USA) for 40 s at speed 6.0. The obtained DNA was purified using the FastDNA SPIN Kit for Soil (MP Biomedicals) according to the manufacturer's protocol. Final aliquots of the total community DNA were further used for PCR-SSCP, 454 pyrosequencing, and qPCR.

### Microbial fingerprinting by PCR-SSCP

Fingerprinting of microbial communities was carried out by PCR-based SSCP described by Schwieger and Tebbe (1998). Bacterial 16S rRNA gene sequences were amplified by PCR using the universal eubacterial primer pair Unibac-II-515f/Unibac-II-927r^P^ (Zachow et al., 2008). The 60 μl reaction mixture contained 1×Taq-&Go Ready-to-use PCR Mix (MP Biomedicals), 3 mM MgCl_2_, 0.2 μM of each primer, and 1 μl DNA template (95°C, 5 min; 32 cycles of 95°C, 20 s; 54°C, 15 s; 72°C, 30 s; and elongation at 72°C, 10 min). For the analysis of the Pseudomonas community, a nested PCR was performed. In the first PCR, the Pseudomonas specific primer pair F311Ps/1459rPs^P^ (Milling et al., 2005) was used in a 20 μl reaction mixture containing 1×Taq-&Go Ready-to-use PCR Mix, 3 mM MgCl_2_, 0.5 mg/ml BSA, 1.5% DMSO, 0.2 μM of each primer, and 1 μl DNA template (94°C, 7 min; 30 cycles of 94°C, 45 s; 56°C, 2 min; 72°C, 2 min; and elongation at 72°C, 10 min). Samples served as templates for the second PCR using the primer pair Unibac-II-515f/Unibac-II-927r^P^. For the analysis of the Firmicutes community, the universal eubacterial primer pair 27f/1492r (Lane, 1991) was used in a 20 μl reaction mixture containing 1×Taq-&Go Ready-to-use PCR Mix, 3 mM MgCl_2_, 0.2 μM of each primer, and 1 μl DNA template (95°C, 5 min; 30 cycles of 95°C, 30 s; 57°C, 30 s; 72°C, 90 s; and elongation at 72°C, 5 min). In the second PCR, the Firmicutes specific primer pair BLS342f/BACr833r^P^ (Blackwood et al., 2005) was used in a 60 μl reaction mixture containing 1×Taq-&Go Ready-to-use PCR Mix, 0.2 μM of each primer, and 3 μl of the product from the first PCR (95°C, 5 min; 30 cycles of 95°C, 45 s; 57°C, 60 s; 72°C, 45 s; and elongation at 72°C, 10 min). The obtained amplicons were separated using the INGENY phorU system (INGENY International BV, Goes, Netherlands) at 400 V and 26°C followed by silver staining. Dominant bands were excised from SSCP gels as described by Schwieger and Tebbe (1998). Extracted DNA fragments were re-amplified by PCR and sequenced. For phylogenetic analysis and identification of related sequences, the obtained sequences were aligned with reference gene sequences from GenBank using BLAST algorithm.

### Amplicon sequencing of bacterial communities

To characterize the rhizosphere bacterial communities associated with *C. recutita* (L.) Rauschert, the V4-V5 hypervariable region of the bacterial 16S rRNA gene (*Escherichia coli* positions 515 to 927) was chosen for the amplification and subsequent pyrosequencing of the PCR products. Due to lack of funds, only samples from the treatments Wb2n-11, Co1-6, Mc5Re-14, P69, and 3Re4-18 from the first sampling time were sequenced. The V4-V5 region was amplified using the primer pair Unibac-II-515f/Unibac-II-927r. The 20 μl reaction mixture contained 1×Taq-&Go Ready-to-use PCR Mix, 3 mM MgCl_2_, 0.5 μM of each primer, and 1 μl of template DNA (95°C, 2 min; 34 cycles of 95°C, 20 s; 65°C, 15 s; 72°C, 29 s; and elongation at 72°C, 10 min). PCR products from four samples of the same treatment were purified with Wizard SV Gel and PCR Clean-Up System (Promega, Madison, WI, USA). Amplicons of each treatment were pooled together in an equimolar ratio and subjected to pyrosequencing using a Roche 454 GS-FLX+ Titanium platform executed by Eurofins MWG (Ebersberg, Germany).

### Processing of pyrosequencing data

Raw sequencing reads were demultiplexed, quality and length filtered using ribosomal database project's (RDP) pyrosequencing pipeline (Cole et al., 2009). Primers were cropped and all sequence reads shorter than 150 bp—with a minimum average quality score <20 and with any ambiguous characters were discarded. Data were normalized to the same number of sequences using an in-house developed Perl script (10 times random re-samplings followed by subset formation (Bragina et al., 2013). A further downstream analysis of normalized data was achieved using the QIIME toolkit (Caporaso et al., 2010). Bacterial sequences were clustered into OTUs using 3, 5, and 20% dissimilarity thresholds with UCLAST (Edgar, 2010), and the most abundant sequence from each OTU was selected as a representative sequence for that OTU. Taxonomy was assigned by using a QIIME-based wrapper of the RDP classifier program (Wang et al., 2007) against the RDP core set (Cole et al., 2009) using an 80% confidence threshold for taxonomic assignment. Rarefaction analysis and estimation of alpha-diversity was performed using Chao1, Shannon, and observed OTU metrics at 3, 5, and 20% dissimilarity. Beta-diversity was examined using weighted UniFrac distances (Lozupone and Knight, 2005) between samples sub-sampled 20 times, with replacement, at a depth of 100 sequences per sample. This method takes phylogenetic relationships between community members in account, incorporating the abundances of phylotypes into the pairwise community comparisons (Eilers et al., 2010). The compositional similarity of all samples was visualized in a three-dimensional principal coordinate system (PCoA) based on previously calculated jackknifed principal coordinates. To reveal the most abundant taxa in different areas of the PCoA plot, taxonomic classification of 20% genetic distance was included.

### Quantitative polymerase chain reaction (qPCR)

The same region of the 16S rRNA gene was amplified by quantitative PCR to determine the total bacterial as well as the Firmicutes abundances in the rhizosphere of *C. recutita* (L.) Rauschert. For the total bacteria, the universal eubacterial primer pair Unibac-II-515f/Unibac-II-927r was used, while the Firmicutes specific primer pair BLS342f/BACr833r was used for Firmicutes. To estimate bacterial gene abundances, standard curves were generated using 10-fold serial dilutions of plasmid DNA containing a full-length copy of either the *P. polymyxa* PB71 16S rRNA gene or the *B. subtilis* Sd3-12 16S rRNA gene (Köberl et al., 2011). For the total bacteria, the qPCR 10 μl reaction mixture contained 1x KAPA SYBR FAST qPCR MasterMix Universal (PEQLAB, Polling, Austria), 0.25 μM of each primer, and 1 μl of the standard and DNA template (95°C, 5 min; 35 cycles of 95°C, 20 s; 54°C, 15 s; 72°C, 30 s; and melt from 72 to 95°C). For Firmicutes, the 10 μl qPCR reaction mixture contained 1x KAPA SYBR FAST qPCR MasterMix Universal, 0.25 μM of each primer, and 1 μl standard and DNA template (95°C, 5 min; 30 cycles of 95°C, 45 s; 56°C, 60 s; 72°C, 45 s; and melt from 72 to 95°C). qPCR was performed in duplicate for each sample using the Rotor-Gene 6000 real-time rotary analyser (Corbett Research, Sydney, Australia). The melting curve analysis of the PCR products was performed immediately after the amplification. Bacterial copy numbers for each reaction were generated from the standard curves and calculated to copy number per g root fresh weight (fw). Each replicate was analyzed two times in two independent runs.

### Confocal laser scanning microscopy (CLSM)

DsRed2-labeled bacteria (3Re4-18) were grown in 5 ml nutrient broth (NB) supplemented with tetracycline (40 μ g ml^−1^) at 30°C and 120 rpm for 24 h. Bacterial cells were collected by centrifugation at 13,500 g for 5 min and resuspended in fresh NB medium without addition of antibiotics. The cell suspension was adjusted to an optical density corresponding to a cell count of 10^9^ cells ml^−1^. Seeds were mixed with 1 ml of the cell suspension in 1.5 ml Eppendorf tubes and incubated at room temperature for 15 min, followed by a washing step with sterile NaCl solution (0.85%). Seeds were placed on a filter paper in moist chambers which were kept at 22°C for 5 days (16/8 h day/night). Seeds incubated with sterile water served as control. Roots of chamomile were examined on a Leica TCS SPE confocal scanning microscope (Leica Microsystems GmbH, Wetzler, Germany).

### Statistics

Computer-assisted comparisons of SSCP generated community profiles were performed using GelComparII (version 5.1, Applied Maths, Kortrijk, Belgium). For the cluster analysis, similarity matrices based on Pearson's correlation coefficients were constructed, and a dendrogram using the unweighted paired group means algorithm (UPGMA) was created. Relative positions and intensity of DNA bands were used for a principal component analysis (PCA). Significances in the difference between the treatments were calculated using PASW Statistics 18 (SPSS Inc., Chicago, IL, USA). First, data were checked for normal distribution and homogeneity of variance. Second, an one-way ANOVA analysis was performed with data that follow a normal distribution, and a *post-hoc* test was applied depending on the homogeneity of the variance index using the Tukey honestly significant difference (HSD) analysis for qPCR data and the Tamhane T2 test for HPLC-MS data.

## Author contributions

Conceived and designed the experiments: Ruth Schmidt, Martina Köberl, Marlene Monschein, Gabriele Berg, Rudolf Bauer, and Elshahat M. Ramadan. Performed the experiments: Ruth Schmidt, Amr Mostafa, Martina Köberl, and Marlene Monschein. Analyzed the data: Ruth Schmidt, Martina Köberl, Marlene Monschein, and Kenneth B. Jensen. Contributed reagents/materials/analysis tools: Gabriele Berg. Wrote the paper: Ruth Schmidt and Gabriele Berg.

### Conflict of interest statement

The authors declare that the research was conducted in the absence of any commercial or financial relationships that could be construed as a potential conflict of interest.
