# Supplementary material for: Effects of bacterial inoculants on the indigenous microbiome and secondary metabolites of chamomile plants
Source: Front Microbiol. 2014 Feb 19;5:64. doi: 10.3389/fmicb.2014.00064 (PMC3928675; doi:10.3389/fmicb.2014.00064)
Supplement: Supplementary file 1 [file DataSheet1.DOCX]

**Supplementary information**

#### Table S1 Content (%) of apigenin-7-*O*-glucside in *Chamomilla recutita* (L.) Rauschert samples.

| **Sample** | **Percentage (%)** | **Confidence** |
| --- | --- | --- |
| Control (n=10) | 0.86 | ± 0.04 a |
| Wb2n-11 (n=10) | 0.87 | ± 0.09 a |
| Co1-6 (n=10) | 1.06 | ± 0.07 b |
| Mc5Re-14 (n=8) | 1.04 | ± 0.06 b |
| L13-6-12 (n=7) | 0.79 | ± 0.06 a |
| P69 (n=10) | 0.78 | ± 0.15 a |
| 3Re4-18 (n=10) | 0.82 | ± 0.06 a |

Statistically significant differences (p<0.05) are marked with different letters.

n = number of samples

#### Table S2 Content (%) of apigenin in *Chamomilla recutita* (L.) Rauschert samples.

| **Sample** | **Percentage (%)** | **Confidence** |
| --- | --- | --- |
| Control (n=10) | 0.94 | 0.10 a |
| Wb2n-11 (n=10) | 0.78 | 0.08 a |
| Co1-6 (n=10) | 0.95 | 0.14 a |
| Mc5Re-14 (n=10) | 0.95 | 0.10 a |
| L13-6-12 (n=10) | 0.84 | 0.07 a |
| P69 (n=10) | 0.77 | 0.12 a |
| 3Re4-18 (n=10) | 0.91 | 0.10 a |

Statistically significant differences (p<0.05) are marked with different letters.

n = number of samples

#### Table S3 Sequencing results of 16S rRNA gene fragments derived from selected SSCP bands using universal eubacterial primers.

| **Band** | **Closest NCBI database match** | **Accession No.** | **Similarity (%)** |
| --- | --- | --- | --- |
| 1U-a | *Bacillus endophyticus* strain p18_C12 | JQ833589.1 | 93 |
| 1U-b | *Serratia marcescens* strain MUD-MaC2 | JX534512.1 | 90 |
| 1U-c | *Pseudomonas psychrotolerans* strain: C36 | NR_042191.1 | 97 |
| 1U-d | No match | - | - |
| 1U-e | *Bacillus flexus* strain p2_D03 | JQ829837.1 | 90 |
| 1U-f | *Klebsiella pneumoniae* strain p95_F10 | JQ830639.1 | 90 |
| 1U-g | *Pantoea dispersa* strain p91_H01 | JQ829612.1 | 93 |
| 1U-h | *Pantoea dispersa* strain p91_H01 | JQ829612.1 | 90 |
| 2U-a | *Flavobacterium flevense* strain: NBRC 14960 | AB680723.1 | 99 |
| 2U-b | *Bacillus selenatarsenatis* strain A1-37c-12 | JX517224.1 | 99 |
| 2U-c | *Bacillus sp.* SG22 | JX402437.1 | 99 |
| 2U-d | *Bacillus cereus* strain p44_E01 | JQ832456.1 | 99 |
| 2U-e | *Anoxybacillus sp.* D1021 | EU926955.1 | 99 |
| 2U-f | *Bacillus sp.* SG22 | JX402437.1 | 100 |
| 2U-g | *Bacillus sp.* S3-R6TC-BA1 | GU325806.1 | 95 |
| 2U-h | *Bacillus sp.* S3-R6TC-BA1 | GU325806.1 | 96 |

#### Table S4 Sequencing results of 16S rRNA gene fragments derived from selected SSCP bands using *Pseudomonas*-specific primers.

| **Band** | **Closest NCBI database match** | **Accession No.** | **Similarity (%)** |
| --- | --- | --- | --- |
| 1P-a | *Pseudomonas sp.* MOC14 | JX122114.1 | 100 |
| 1P-b | *Pseudomonas sp.* MOC14 | JX122114.1 | 100 |
| 1P-c | *Pseudomonas mandelii* strain BJC15-C35 | JX469391.1 | 100 |
| 1P-d | *Dokdonella sp.* CC-YHH031 | GQ281768.1 | 97 |
| 1P-e | *Pseudomonas sp.* OF38 | HM626451.1 | 99 |
| 1P-f | *Xanthomonadaceae* bacterium K-1-9 | JQ963326.1 | 97 |
| 1P-g | *Lysobacter sp.* 7C-9 | JQ349048.1 | 97 |
| 2P-a | *Pseudomonas sp.* MOC14 | JX122114.1 | 100 |
| 2P-b | *Pseudomonas sp.* MOC14 | JX122114.1 | 100 |
| 2P-c | *Lysobacter sp*. 9NM-14 | JQ608331.1 | 100 |
| 2P-d | *Pseudomonas fluorescens* strain BIM B-187 | GU784932.1 | 99 |
| 2P-e | *Lysobacter sp.* ljh-29 | GU217698.1 | 99 |
| 2P-f | *Pseudomonas mandelii* strain BJC15-C35 | JX469391.1 | 99 |
| 2P-g | *Pseudomonas sp.* MOC14 | JX122114.1 | 99 |

####

#### Table S5 Sequencing results of 16S rRNA gene fragments derived from selected SSCP bands using *Firmicutes*-specific primers.

| **Band** | **Closest NCBI database match** | **Accession No.** | **Similarity (%)** |
| --- | --- | --- | --- |
| 1F-a | *Bacillus sp.* BMR7 | JX434152.1 | 99 |
| 1F-b | *Bacillus sp.* BMR7 | JX434152.1 | 100 |
| 1F-c | *Bacillus korlensis* strain A2-37c-4 | JX517217.1 | 99 |
| 1F-d | *Bacillus sp.* DV9-6 | GQ407151.1 | 99 |
| 1F-e | *Bacillus megaterium* strain BS2 | JX455196.1 | 100 |
| 1F-f | *Bacillus sp.* DV9-6 | GQ407151.1 | 99 |
| 2F-a | *Bacillus sp.* BMR7 | JX434152.1 | 99 |
| 2F-b | *Bacillus sp.* NMLR7 | JX434112.1 | 98 |
| 2F-c | *Bacillus sp.* D22-4C | EU931539.1 | 98 |
| 2F-d | *Bacillus sp.* DV9-6 | GQ407151.1 | 99 |
| 2F-e | *Bacillus flexus* strain TAX4 | JX280923.1 | 98 |
| 2F-f | *Bacillus sp.* SCSIO 15042 | JX232168.1 | 99 |

####
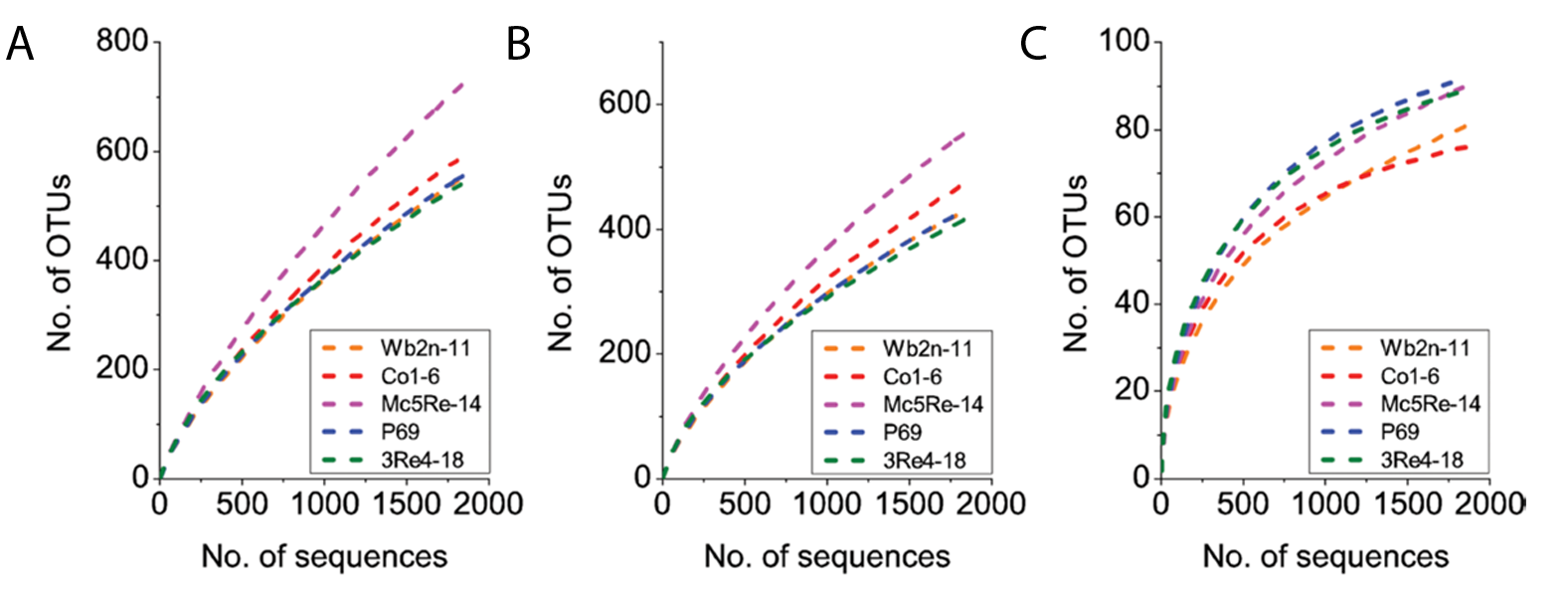


Figure S1 Rarefaction analysis of the 16S rRNA gene amplicon libraries of rhizosphere samples of *Chamomilla recutita* (L.) Rauschert. Rarefaction curves were calculated at (A) 3%, (B) 5% and (C) 20% genetic distance levels, corresponding to the taxonomic levels of species, genera and phyla, respectively. Coloured lines depict different treatments.


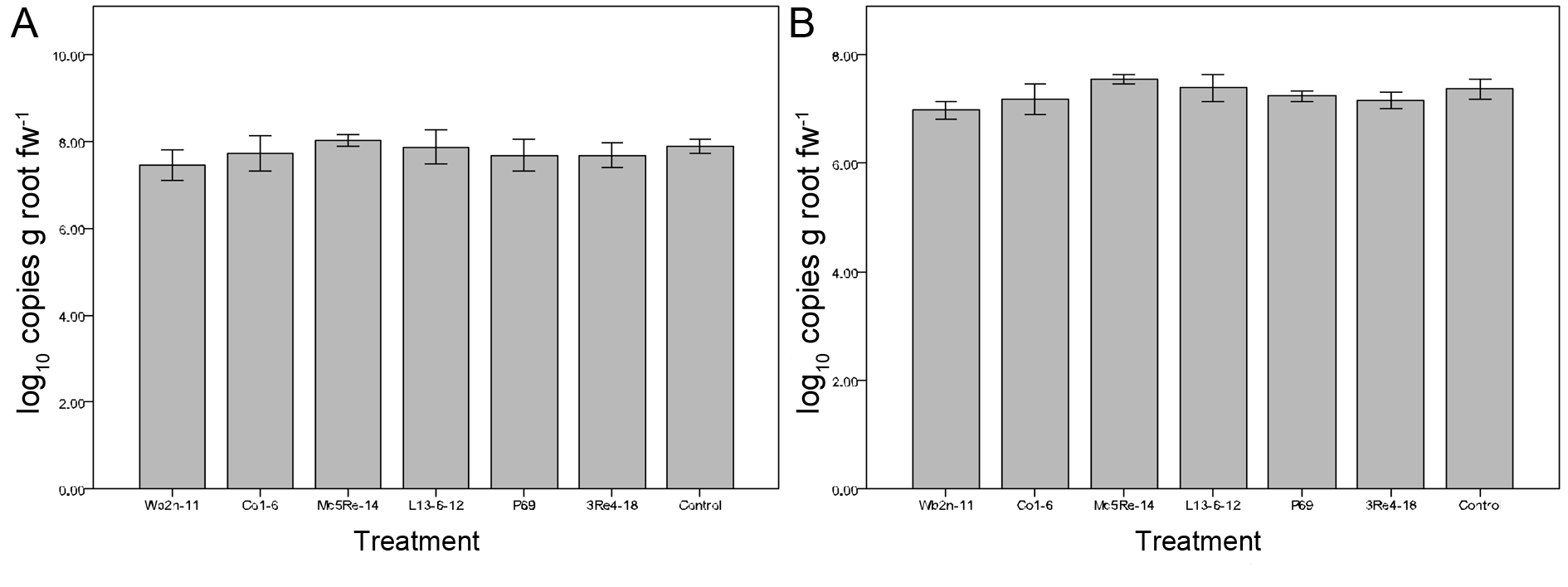


Figure S2 Abundances of (A) total bacteria and (B) *Firmicutes* obtained by qPCR of the 16S rRNA genes. Averages of 16S rRNA gene copy numbers per gram root fresh weight as log_10_ and confidences are shown.
